# Selenium-modified hydroxyapatite titanium coating: enhancing osteogenesis and inhibiting cancer in bone invasion by head and neck squamous cell carcinoma

**DOI:** 10.3389/fbioe.2025.1552661

**Published:** 2025-02-24

**Authors:** Xutao Wen, Qin Zhou, Sihan Lin, Huaming Mai, Ling Zhang

**Affiliations:** ^1^ Department of Oral and Maxillofacial-Head and Neck Oncology, Shanghai Ninth People’s Hospital, Shanghai Jiao Tong University School of Medicine, Shanghai, China; ^2^ Shanghai Key Laboratory of Stomatology, National Center for Stomatology, National Clinical Research Center for Oral Diseases, College of Stomatology, Shanghai Research Institute of Stomatology, Shanghai Jiao Tong University, Shanghai, China; ^3^ Shanghai Engineering Research Center of Advanced Dental Technology and Materials, Shanghai Key Laboratory of Stomatology, Shanghai Research Institute of Stomatology, Shanghai, China; ^4^ Department of Oral and Maxillofacial Surgery, College and Hospital of Stomatology, Guangxi Medical University, Nanning, China; ^5^ Department of Oral Surgery, Shanghai Ninth People’s Hospital, Shanghai Jiao Tong University School of Medicine, Shanghai, China; ^6^ Department of Prosthodontics, Shanghai Ninth People’s Hospital, Shanghai Jiao Tong University School of Medicine, Shanghai, China; ^7^ Department of Oral and Maxillofacial Surgery, Kashgar Prefecture Second People’s Hospital, Xinjiang, China

**Keywords:** antitumor, osteogenesis, titanium, micro-arc oxidation, selenium

## Abstract

**Introduction:**

Head and neck squamous cell carcinoma (HNSCC) frequently invades the jaw, and surgical treatment often leads to bone defects requiring reconstruction with titanium plates. To enhance the anti-tumor and bone regeneration properties of titanium, a selenium-modified hydroxyapatite coating was developed on titanium surfaces.

**Methods:**

Selenium-modified hydroxyapatite coatings was fabricated using micro-arc oxidation (MAO). The coating properties were characterized by SEM, XPS, AFM, Contacting angle test and ICP-OES. Cell proliferation assays were performed using rBMSCs and Cal27 cells. The osteogenic potential of the materials was assessed via ALP and OCN immunofluorescence staining and quantitative polymerase chain reaction (qPCR). Apoptosis in Cal27 cells was analyzed through flow cytometry, and ROS levels in rBMSCs and Cal27 cells were measured using ROS fluorescent probes.

**Results:**

A coating was successfully formed on the surface of titanium with a porous structure via MAO. The atomic percentages of calcium, phosphorus and selenium on the coating surface were 42.47%, 45.43% and 12.3%, respectively, and the ion components could be released steadily and slowly. *In vitro*, 0.2 µg/mL selenium had toxic effects on Cal27 and promoted osteogenic differentiation of rBMSCs. PCR showed that selenium increased the expression of genes related to osteogenic differentiation of rBMSCs by 3–5 times. ROS detection found differences in intracellular ROS content between Cal27 and rBMSCs.

**Discussion:**

By incorporating selenium-modified coatings, titanium implant materials can simultaneously promote osteogenesis and inhibit tumor growth, offering a promising strategy for postoperative functional recovery in HNSCC patients.

## 1 Introduction

Head and neck squamous cell carcinoma (HNSCC) is one of the most prevalent malignant tumors, characterized by aggressive and invasive growth (Chuang et al., 2008; Buim et al., 2010; Li et al., 2023). Statistics indicate that approximately half of HNSCC cases involve invasion into adjacent jaw tissue (Brown et al., 2002; Jo et al., 2017). Currently, surgery remains the primary treatment for HNSCC, frequently requiring extensive resection of the tumor and affected jaw (Chamoli et al., 2021). However, jaw resection not only significantly compromises the patient’s quality of life but is also associated with a poor prognosis. Studies have shown that the survival rate of patients with jaw invasion after surgery is markedly lower than that of patients without bone invasion (DeAngelis et al., 2019). The free fibular flap has become the standard of care, and its combination with bridging plates effectively restores jaw morphology (Kammerer and Al-Nawas, 2023). Nevertheless, it is associated with certain risks, including prolonged operation duration and complex preparation processes, and while it primarily addresses bone defect repair, a considerable risk of recurrence persists following tumor resection. Consequently, researchers have begun to investigate biomaterials capable of simultaneously repairing bone defects and inhibiting tumor recurrence.

Hydroxyapatite (HA) is the primary inorganic component of bones and teeth, recognized for its superior biocompatibility and bioactivity compared to other materials (Fendi et al., 2024). HA surface modification of prosthetic metal implants is widely used to enhance bone stability (Zhang et al., 2019; Popkov et al., 2023). Notably, HA can be functionalized with ion substitutions to improve its reparative properties (Salam and Gibson, 2022). For instance, doping with magnesium, strontium, or zinc ions enhances bone regeneration (Hou et al., 2023). The incorporation of silver ions imparts localized antimicrobial capabilities (Ivankovic et al., 2023; Piecuch et al., 2023). Similarly, the addition of cerium ions provides effective anti-inflammatory and antioxidative effects (Kim and Kim, 2022). Recent studies have demonstrated that introducing anionic selenium into HA can effectively inhibit osteosarcoma (Barbanente et al., 2021; Huang et al., 2024). While selenium frequently exerts an inhibitory effect on cancer when used in conjunction with chemotherapeutic agents, often resulting in a systemic response (Zhang et al., 2024). Thus, selenium-modified HA coatings on titanium implants not only inhibit HNSCC, but also achieve the effect of reducing systemic reactions. This approach offers a promising strategy for restoring bone continuity and reducing local recurrence in postoperative HNSCC patients.

Micro-arc oxidation (MAO) enhances the biocompatibility of materials by applying high voltage to induce an oxidation reaction on the anodized metal surface, forming a ceramic-like oxide film (Sheng et al., 2022). Our previous studies successfully utilized MAO to create ceramic coatings on titanium surfaces incorporating magnesium and zinc ions (Li et al., 2020a; Zhou et al., 2022; Yu et al., 2023). Historically, MAO research has primarily focused on the incorporation of specific cations into metal surfaces, with relatively limited exploration of anion incorporation. In this study, selenite was successfully introduced into the titanium surface coating by optimizing the electrolyte composition and instrument parameters, enabling an investigation of its osteogenic and anti-tumor properties.

To our knowledge, no experimental studies have investigated selenium-doped hydroxyapatite for head and neck squamous cell carcinoma (HNSCC), nor has there been substantial research on incorporating anions into titanium surface coatings via micro-arc oxidation (MAO). In this study, selenite was successfully incorporated into a porous hydroxyapatite coating on titanium using MAO. The resulting selenium-modified coating exhibited excellent structural properties and biocompatibility, effectively inhibiting HNSCC growth and promoting bone regeneration. Using human tongue squamous cell carcinoma cell lines and rat mesenchymal stem cells, the anti-tumor and osteogenic effects were further explored *in vitro*, offering a promising material for postoperative repair in patients with jaw invasion by HNSCC.

## 2 Materials and methods

### 2.1 Sample and coating preparation

Round pure titanium plates (10 mm in diameter, 1 mm thick, TA1) were used as substrates for the porous coating. The oxide layer on the titanium surface was removed using abrasive paper, followed by sequential ultrasonic cleaning with acetone, anhydrous ethanol, and deionized water for 5 min each. For the experimental group (SeHAMAO), the electrolyte consisted of 0.05 mol/L calcium acetate monohydrate (C_4_H_6_CaO_4_·H_2_O, Aladdin, Shanghai, China), 0.02 mol/L sodium β-glycerophosphate pentahydrate (C_3_H_7_Na_2_O_6_P·5H_2_O, Aladdin, Shanghai, China), and 0.01 mol/L sodium selenite (Na_2_SeO_3_, Aladdin, Shanghai, China). The negative control group (HAMAO) used the same electrolyte without sodium selenite. Porous ceramic coatings were applied to the titanium plates using micro-arc oxidation (MAO, WHD-20, Harbin, China). The MAO process was performed under a constant current of 0.8 A for 8 min, with a pulse frequency of 1,000 Hz and a duty cycle of 10%. After treatment, the samples were rinsed with deionized water and air-dried.

### 2.2 Characterization

The surface morphology and elemental distribution of the coatings were analyzed using scanning electron microscopy (SEM, ZEISS Gemini SEM 300, Germany). The chemical compositions of the HAMAO and SeHAMAO coatings were determined by X-ray photoelectron spectroscopy (XPS, Thermo Fisher Nexsa, United States). Ionic components of the coatings and electrolytes were quantified using inductively coupled plasma optical emission spectroscopy (ICP-OES, Agilent 5,110, United States). The wetting properties of the coating surfaces were measured with a contact angle meter (SDC-350KS, China), and surface roughness was evaluated using atomic force microscopy (AFM, Bruker Dimension Icon, Germany).

### 2.3 Cell culture of rBMSCs and Cal27

Rat bone marrow mesenchymal stem cells (rBMSCs) were isolated and purified from the long bone marrow of 3-week-old Sprague Dawley (SD) rats. The SD rats were sourced from the Animal Centre of the Ninth People’s Hospital of Shanghai Jiao Tong University, with all procedures approved by the institutional animal ethics committee. Second to fourth passage rBMSCs were used for subsequent experiments. Both Cal27 cells and rBMSCs were cultured and expanded in Dulbecco’s Modified Eagle’s Medium (DMEM, Gibco, United States) supplemented with 10% fetal bovine serum (FBS) and 1% penicillin/streptomycin (Gibco, United States).

### 2.4 Preparation of HAMAO and SeHAMAO extract

Ion Release: Titanium plates from the HAMAO and SeHAMAO groups were immersed in equal volumes (5 mL) of deionized water and placed in a 37°C shaker at 75 rpm. The solution was extracted on days 1, 3, 5, and 7. After each extraction, an equal volume of fresh deionized water was added to continue the immersion process. The concentrations of Ca, P, and Se in the extracts were analyzed using inductively coupled plasma optical emission spectroscopy (ICP-OES).

Selenium-Containing Extract: Titanium plates from the HAMAO and SeHAMAO groups were immersed in 5 mL of Dulbecco’s Modified Eagle’s Medium (DMEM) and placed in a 37°C shaker at 75 rpm for 1 day. The original extract, referred to as “1,” was collected and divided into 20 aliquots for subsequent experiments.

### 2.5 Live/dead fluorescence staining and cell counting Kit-8 (CCK-8) assay

To evaluate the effects of the coatings on Cal27 and rBMSCs, 2.5 × 10^5^ Cal27 cells and 5 × 10^4^ rBMSCs were seeded onto the titanium plates from each group. After 24 h of incubation, cells were treated with a Calcein/PI Live/Dead Viability/Cytotoxicity Assay Kit (Beyotime, China) and observed using confocal laser scanning microscopy (CLSM, Leica, Germany).

To determine the optimal synergistic concentrations of the extracts *in vitro*, 5 × 10^4^ Cal27 cells and 1 × 10^4^ rBMSCs were seeded into 96-well plates and treated with different concentrations of the extracts, divided into 20 groups. After 24 h, the medium was mixed with CCK-8 reagent (DOJINDO, Japan) in a 10:1 ratio, added to the wells, and incubated for 2 h. Absorbance was measured at 450 nm using a microplate reader. All experiments were performed in triplicate.

To explore the time-dependent effects of the extracts, equal concentrations of HAMAO and SeHAMAO extracts were applied to Cal27 cells for 6, 12, 18, 24, and 48 h. Cell viability was then assessed using the CCK-8 assay.

### 2.6 Alkaline phosphatase (ALP) staining

The osteogenic properties of the coating materials were evaluated using alkaline phosphatase (ALP) staining. Third-passage (P3) rBMSCs were seeded onto the surfaces of three sets of titanium slices, with fresh medium replaced regularly. In parallel, rBMSCs were treated with extracts under the same conditions. After 5 days, the culture medium was aspirated, and the samples were washed with phosphate-buffered saline (PBS) and fixed in 4% paraformaldehyde for 30 min. Following fixation, the samples were washed again with PBS. ALP staining solution was then added, and the samples were incubated for 30 min. After the staining solution was aspirated, the samples were washed with PBS, and the reaction was terminated by placing them on a shaker with gentle shaking for 3 min. Finally, the stained rBMSCs were photographed to observe ALP activity.

### 2.7 Immunofluorescence staining

The osteogenic ability of the extract was examined by immunofluorescence staining with ALP (RD, AF2910, United States) and OCN (RD, MAB1419, United States). rBMSCs were inoculated at a density of 5 × 10^4^ in 35 mm confocal dishes and the solution was changed every 2 days. After 5 days, the culture was aspirated and discarded, washed with phosphate buffer, and fixed for 0.5 h by adding a fixative. And then, 0.5% Triton (Sigma, United States) was permeabilized at room temperature for 20 min, followed by adding 5% fetal bovine serum for 2 h. The primary antibody was added and placed in the refrigerator at four degrees overnight. The fluorescent secondary antibody (Alexa Fluor 594, 1: 200, Yeasen, Shanghai, China) was added the next day and protected from light for 1 h at room temperature. Finally, FITC (Yeasen, Shanghai, China) staining and DAPI (Yeasen, Shanghai, China) staining were performed to determine the cytoskeletal morphology and cytosolic status of rBMSCs. Follow-up fluorescence photography was performed using a laser scanning confocal microscope (Leica, Germany).

### 2.8 Osteogenic gene expression

rBMSCs were seeded into 6-well plates at a density of 2 × 10^5^ cells/well and allocated into four groups according to the culture medium: DMEM, osteogenic medium (OM), HAMAO extract (ex-HAMAO), and SeHAMAO extract (ex-SeHAMAO). Fresh medium was replaced regularly. After 5 days of culture, the expression levels of osteogenesis-related genes in each group of rBMSCs were analyzed using quantitative PCR (qPCR). The primer sequences used in these experiments are listed below: rGAPDH forward: CAG​GGC​TGC​CTT​CTC​TTG​TG, reverse: AAC​TTG​CCG​TGG​GTA​GAG​TC; rBmp2 forward: ACC​GTG​CTC​AGC​TTC​CAT​CAC, reverse: TTC​CTG​CAT​TTG​TTC​CCG​AAA; rCol1 forward: CAT​GTT​CAG​CTT​TGT​GGA​CCT, reverse: GCA​GCT​GAC​TTC​AGG​GAT​GT; rOPN forward: GAG​GAG​AAG​GCG​CAT​TAC​AG, reverse: ATG​GCT​TTC​ATT​GGA​GTT​GC; rRUNX2 forward: CCT​TCC​CTC​CGA​GAC​CCT​AA, reverse: ATG​GCT​GCT​CCC​TTC​TGA​AC.

### 2.9 Flow cytometry of Cal27

Cal27 cells were seeded into 6-well plates at a density of 2 × 10^5^ cells/well and divided into three groups: DMEM, ex-HAMAO, and ex-SeHAMAO. After 48 h of culture, cell viability was assessed by flow cytometry using an Annexin V-FITC Apoptosis Detection Kit (BD Biosciences, United States), following the manufacturer’s instructions. In parallel, Cal27 cells were seeded into confocal culture dishes at a density of 1 × 10^4^ cells/dish. After 48 h of culture under the same conditions, the cells were treated as described above and then observed using a laser confocal microscope.

### 2.10 Detection of reactive oxygen species

Cal27 and rBMSCs were seeded into 6-well plates at a density of 2 × 10^5^ cells/well and allocated into four groups: DMEM, ROS, ex-HAMAO, and ex-SeHAMAO. After 48 h of culture, cells were treated with a Reactive Oxygen Species Assay Kit (Yeasen, Shanghai) according to the manufacturer’s instructions, then observed and photographed under a fluorescence microscope. In parallel, Cal27 and rBMSCs were also seeded into 96-well plates (Corning, 3875, United States) at a density of 1 × 10^4^ cells/well. After 48 h, cell viability was assessed using the CCK-8 assay, followed by ROS detection using the ROS Assay Kit. Fluorescence intensity was measured with a microplate reader, and the average intracellular ROS fluorescence intensity was calculated.

### 2.11 Statistical analysis

Data from this study were analyzed using one-way ANOVA in GraphPad Prism to determine statistical significance. ImageJ and Origin software were used to generate figures. Statistical significance levels were set at *P < 0.05, **P < 0.01, ***P < 0.001, and ****P < 0.0001.

## 3 Results

### 3.1 Characterization

**FIGURE 1:**
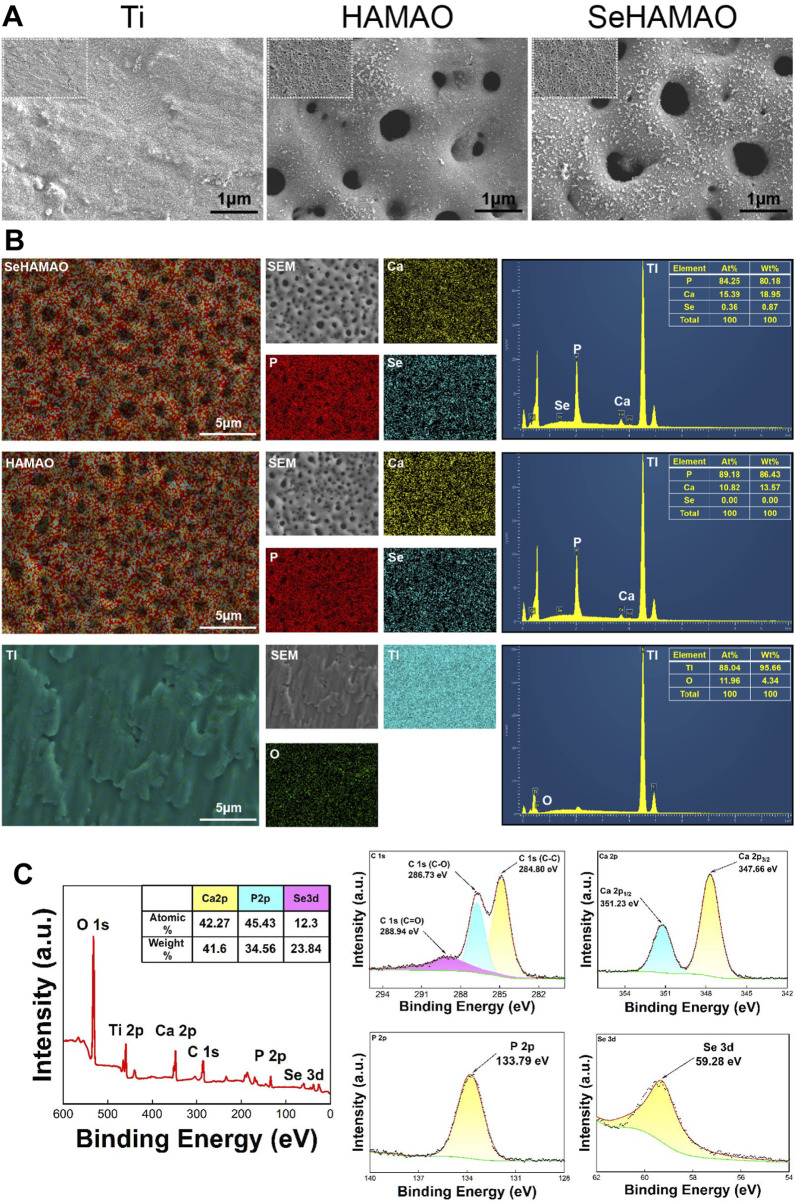
**(A)** Scanning electron microscopy images showing the morphological structures of the three groups of materials. **(B)** Elemental distribution and proportions in the three groups of materials, as determined by EDS. **(C)** X-ray photoelectron spectroscopy (XPS) analysis of the SeHAMAO group, showing the binding energies of each element.

Micro-arc oxidation (MAO) successfully produced a coating on the titanium surface. Among the three groups tested, the two groups subjected to MAO formed a uniformly distributed, micron- and nano-scale porous structure (Figure 1A). Energy-dispersive X-ray spectroscopy (EDS) analysis of the titanium surface and its coatings confirmed the successful incorporation of Ca, P, and Se into the coatings (Figure 1B). In the SeHAMAO group, the primary elemental atomic percentages were approximately Ca 84.25%, P 15.39%, and Se 0.36%. To reduce measurement errors, the elemental composition was further quantified using X-ray photoelectron spectroscopy (XPS), which detected O, Ti, Ca, P, C, and Se in the SeHAMAO group. The three main elements identified by XPS were Ca (42.27%), P (45.43%), and Se (12.3%) (Figure 1C). Analysis of binding energies revealed that C exhibited three peaks at 286.73 eV, 284.80 eV, and 288.94 eV; Ca had two peaks at 347.66 eV and 351.23 eV; P had a single peak at 133.79 eV; and Se had a single peak at 59.28 eV (Figure 1C).

**FIGURE 2:**
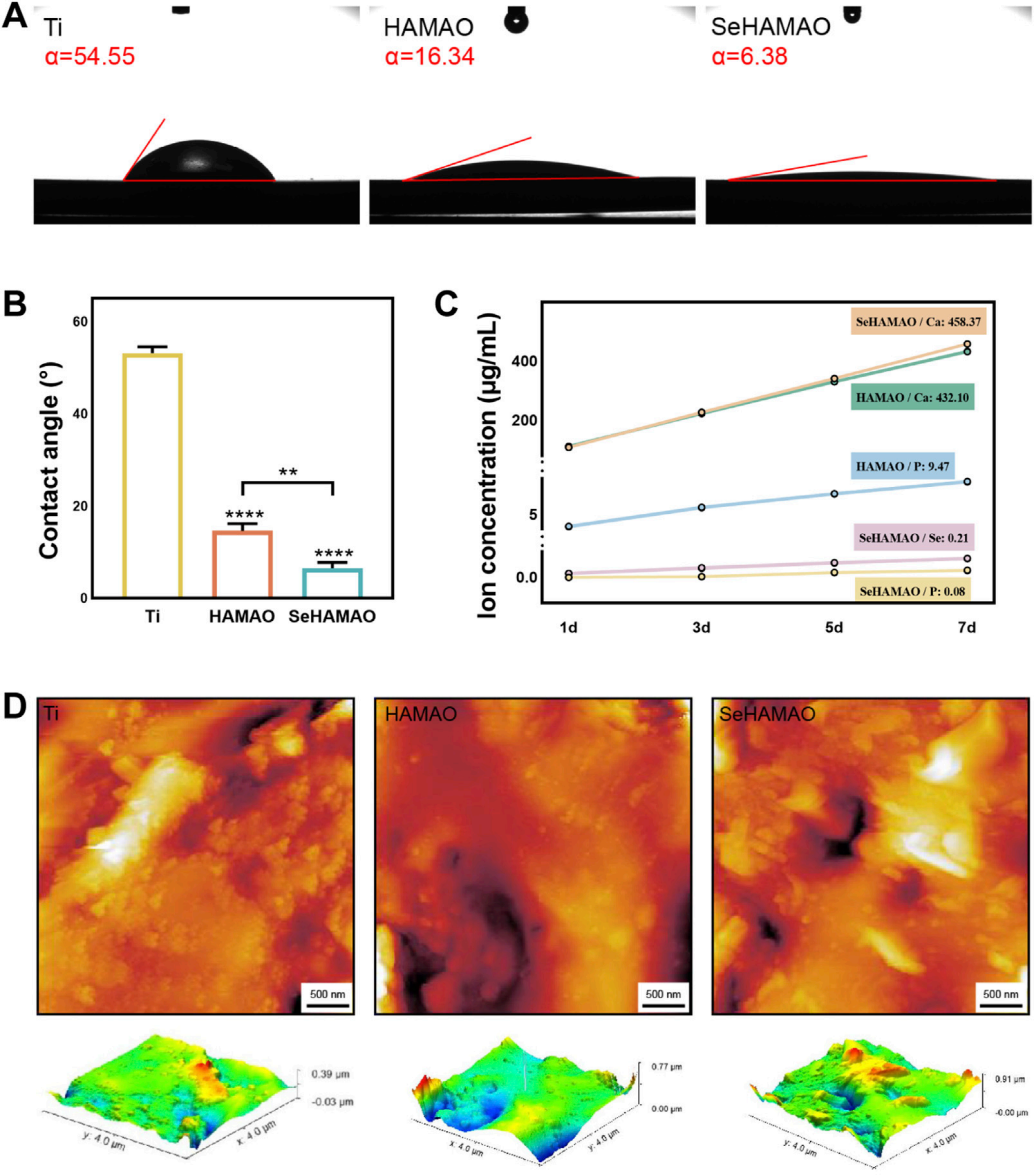
**(A, B)** Contact angles of the four groups of materials. **(C)** Ion release profiles of three groups at 1, 3, 5, and 7 days **(D)** Two-dimensional (2D) and three-dimensional (3D) atomic force microscopy (AFM) images of the three groups. **P < 0.01 and ****P < 0.0001 indicate statistically significant differences.

The contact angles of the Ti, HAMAO, and SeHAMAO groups were 53.17° ± 1.39°, 14.16° ± 1.56°, and 6.59° ± 1.22°, respectively (Figures 2A, B). Compared with the Ti group, the HAMAO-treated group exhibited a smaller contact angle. Moreover, the Se-doped coating (SeHAMAO) demonstrated an even smaller contact angle and stronger hydrophilicity. Ion release measurements showed that Ca, P, and Se concentrations increased linearly and gradually over time, indicating continuous ion release from the coating. After 7 days, the ion concentrations in the SeHAMAO extract were 458.37 μg/mL for Ca, 0.08 μg/mL for P, and 0.21 μg/mL for Se. In contrast, the HAMAO extract contained 432.10 μg/mL of Ca and 9.47 μg/mL of P (Figure 2C). Two-dimensional and three-dimensional atomic force microscopy (AFM) images of the three groups confirmed these findings (Figure 2D).

### 3.2 Cytotoxicity and proliferation

**FIGURE 3:**
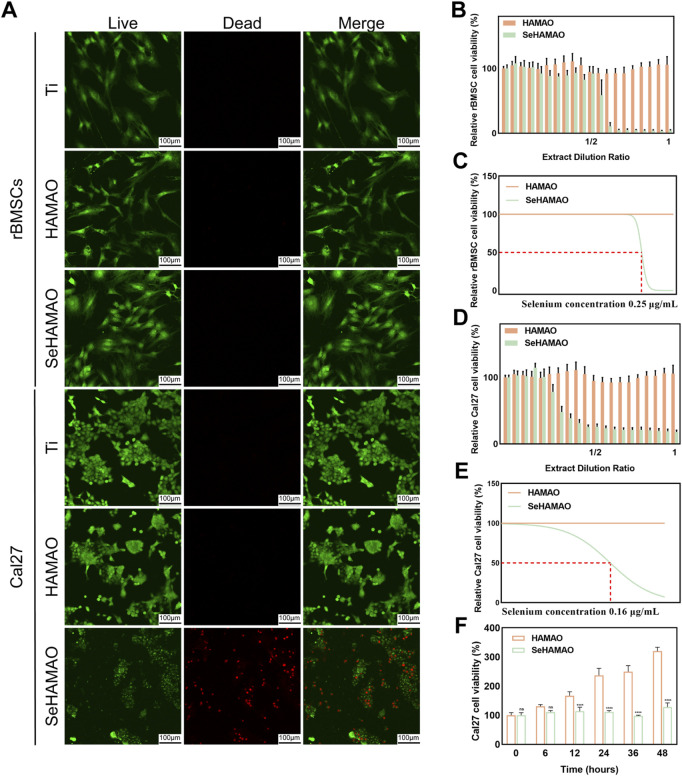
**(A)** Live-dead staining of rBMSCs and Cal27 cells cultured on three different titanium surfaces. **(B)** The effects of various extract concentrations on rBMSC viability. **(C)** Fitting curves and IC₅₀ values for rBMSCs under different extract concentrations. **(D)** The effects of various extract concentrations on Cal27 cell viability. **(E)** Fitting curves and IC₅₀ values for Cal27 cells under different extract concentrations. **(F)** Time-dependent changes in Cal27 cell viability at an extract concentration containing 0.2 μg/mL of Se.

Live-dead cell staining of rBMSCs and Cal27 cells cultured on the three groups of samples revealed that, except for a large number of dead Cal27 cells on the SeHAMAO group, cells in all other groups remained viable (Figure 3A). When treated with the extracts, the CCK-8 proliferation assay demonstrated that extracts containing selenium had a significant inhibitory effect on rBMSCs after surpassing a certain concentration (Figures 3B,C). In contrast, for Cal27 cells, the inhibitory effect increased continuously with the concentration of the selenium-containing extract (Figures 3D,E). Statistical analysis identified a selenium concentration of 0.2 μg/mL as suitable for testing on Cal27 cells. Over a 48-h period, Cal27 cell viability declined with time when compared to the control group (Figure 3F).

### 3.3 Effect of coating surface on osteogenesis

**FIGURE 4:**
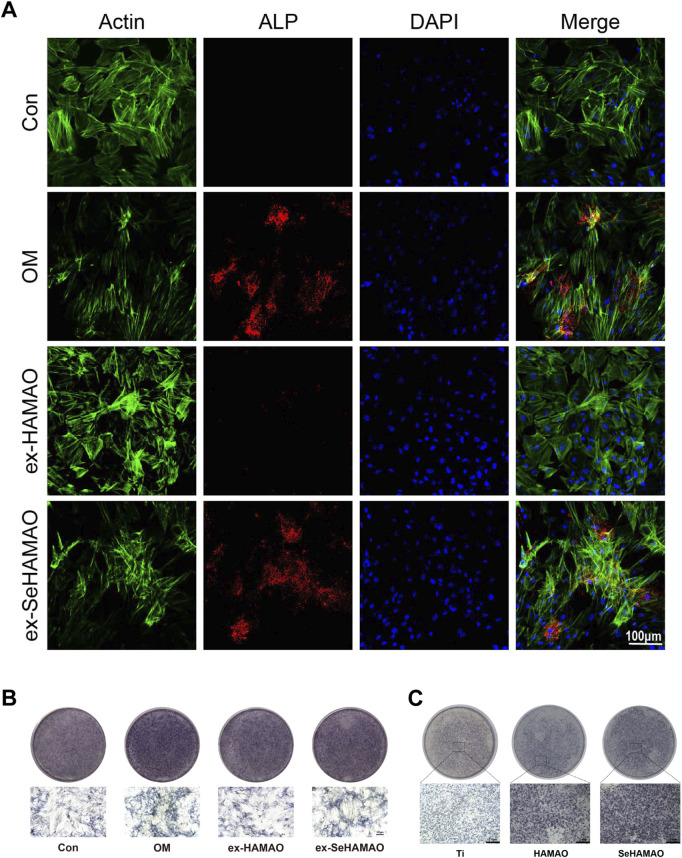
**(A)** Immunofluorescence staining showing ALP expression. **(B)** ALP staining of rBMSCs treated with different media. **(C)** ALP staining of rBMSCs cultured on different substrates.

After confirming that the coating material was non-toxic to rBMSCs, we examined its effects on osteogenic differentiation. Compared with the control, cells treated with the selenium-containing extract showed elevated ALP expression, although this increase was less pronounced than that observed in cells cultured with osteogenic medium (Figures 4A, B). ALP staining of rBMSCs grown on the material’s surface indicated that both HAMAO and SeHAMAO coatings enhanced ALP expression, with the Se-modified coating exerting a stronger effect (Figure 4C).

**FIGURE 5:**
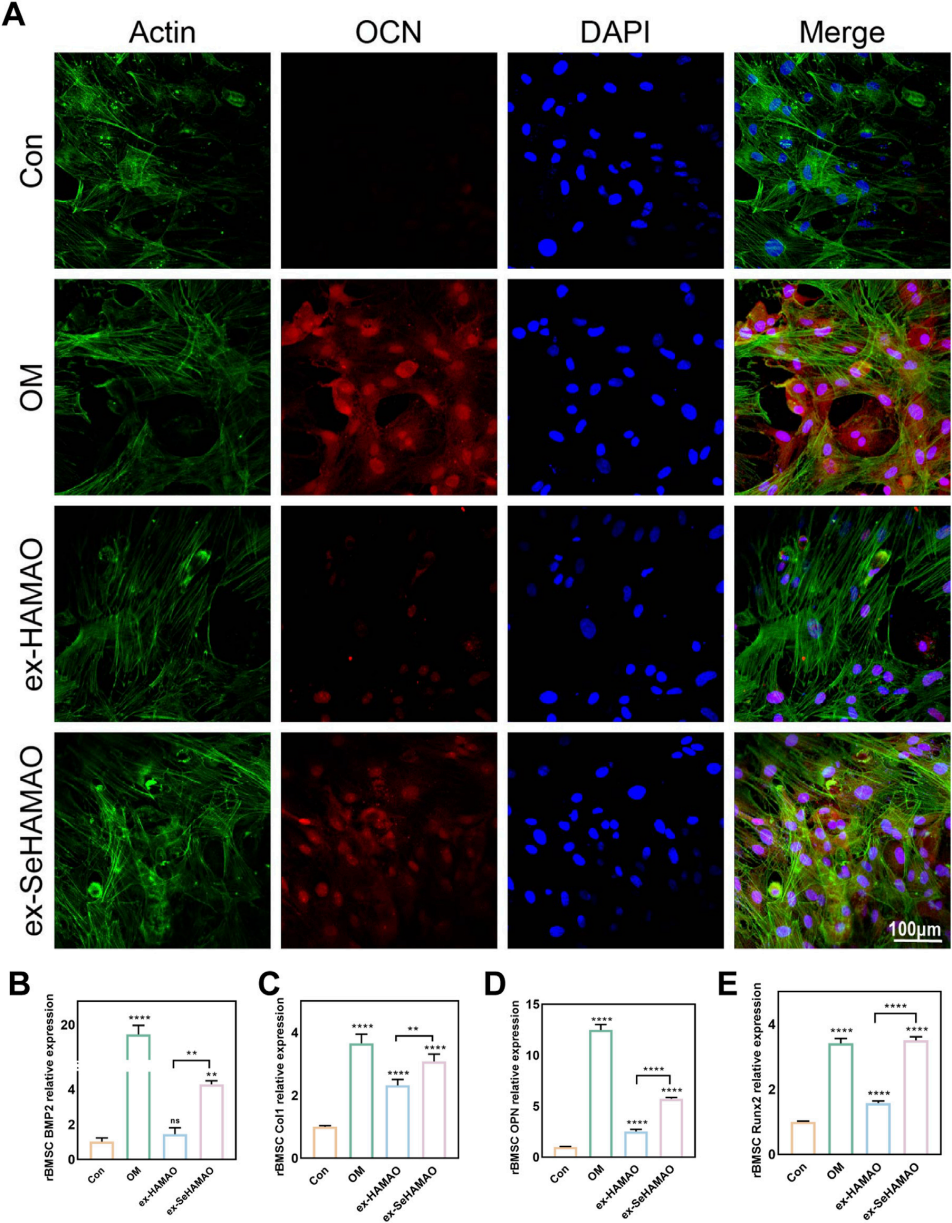
**(A)** OCN expression in rBMSCs as detected by immunofluorescence staining. **(B–E)** Relative expression levels of osteogenesis-related genes (BMP2, Col1, OPN, and Runx2) in rBMSCs. **P < 0.01 and ****P < 0.0001 indicate statistically significant differences, while ns denotes no significant differences between groups.

We next examined OCN expression in rBMSCs. Immunofluorescence staining revealed that OCN expression was significantly enhanced in cells treated with the selenium-containing extract, although it remained lower than that observed in cells cultured with osteogenic medium (Figure 5A). Semi-quantitative gene expression analysis showed that the relative expression levels of BMP2, Col1, OPN, and RUNX2 were all elevated in the selenium-containing extract group (Figures 5B–E).

### 3.4 Reactive oxygen species detection and apoptosis flow cytometry

**FIGURE 6:**
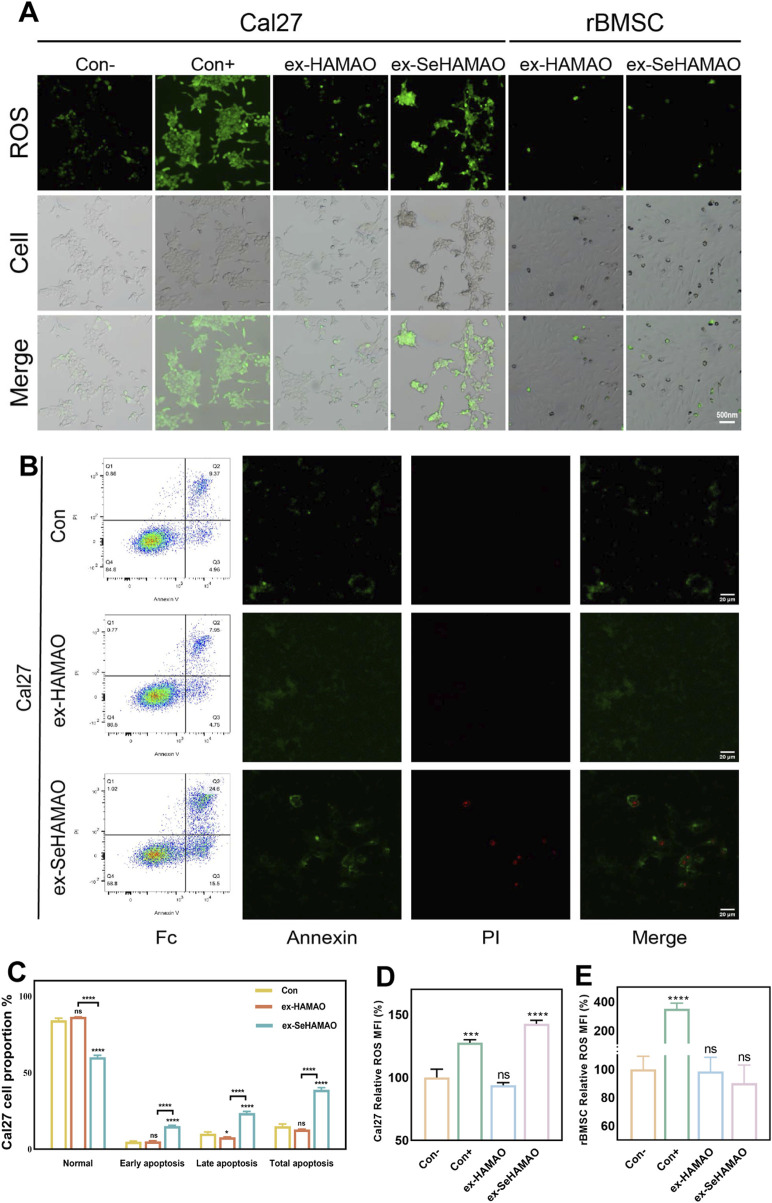
**(A)** Fluorescence detection of intracellular ROS in rBMSCs and Cal27 cells. **(B)** Annexin V-FITC flow cytometry analysis of Cal27 cells, showing the proportion of apoptotic cells and the distribution of intracellular fluorescence. **(C)** Statistical analysis of percentage of apoptotic cells in Cal27. **(D)** Statistical analysis of the mean fluorescence intensity of ROS in Cal27 cells. **(E)** Statistical analysis of the mean fluorescence intensity of ROS in rBMSCs. *P < 0.05 and ****P < 0.0001 indicate statistically significant differences, while ns denotes no significant differences between groups.

To investigate the underlying reasons for the differential effects of the selenium-containing extract on cells, we measured ROS levels in both cell types. Cal27 cells treated with the selenium-containing extract exhibited abnormal cell morphology and increased intracellular ROS levels, whereas no significant changes were observed in the other groups (Figures 6A, D, E). Flow cytometry analysis of apoptosis in Cal27 cells further revealed that the selenium-containing extract led to a marked increase in apoptotic and necrotic cells compared with normal conditions (Figures 6B, C).

## 4 Discussion

Currently, coating modifications on titanium surfaces are predominantly applied in implants, enhancing osseointegration and reducing infection and inflammation (Wu et al., 2021; Costa et al., 2023; Feng et al., 2023) However, in cases of bone defects resulting from maxillofacial tumor surgeries, both bone repair and the prevention of postoperative tumor recurrence are necessary. In recent years, materials with dual antitumor and bone regeneration properties have primarily targeted osteosarcoma (Li et al., 2020b; Jing et al., 2024). Given the epidemiological and structural characteristics of the head and neck, squamous cell carcinoma frequently invades the jaw (Yao, 2020). Therefore, we combined selenium-doped hydroxyapatite, previously shown to be effective against osteosarcoma, with titanium plates to successfully produce a selenium-modified hydroxyapatite titanium coating. Furthermore, we demonstrated its capacity to inhibit head and neck squamous cell carcinoma and promote bone regeneration *in vitro*.

Micro-arc oxidation (MAO) facilitates the incorporation of electrolyte-derived elements into the surface coating of titanium substrates. In this study, sodium selenite was added to the electrolyte. Although flocculation was observed within the solution, we proceeded to analyze the electrolyte composition using inductively coupled plasma-optical emission spectroscopy (ICP-OES), which revealed the following concentrations: Ca 1,600 mg/L, P 421 mg/L, and Se 225 mg/L. Subsequently, the elemental composition present on the titanium surface was examined by X-ray photoelectron spectroscopy (XPS). The binding energy (BE) of the P 2p peak was measured at 133.79 eV, indicating that phosphorus predominantly exists in the form of phosphate. Similarly, the BE of the Se 3d peak, at 59.28 eV, confirmed that selenium is present as selenite (Figure 1C). (Zhang et al., 2017; Chubar, 2023)

MAO can create a multi-porous coating on the titanium surface, facilitating cell adhesion (Figure 2D). When we seeded cells on the coated material, the SeHAMAO group showed greater cytotoxicity towards Cal27 cells. Considering that rBMSCs are of mesenchymal origin while Cal27 cells are epithelial in origin, their structures and morphologies differ. To exclude the influence of the porous structure on the cells, we applied the material extract directly and obtained similar results. Thus, we believe that selenite plays a crucial role in tumor suppression. Consistent with previous findings, selenite exerts significant antitumor effects, including reduced cancer incidence, inhibition of tumor invasion and metastasis, and potential clinical applications in combination with radiation and chemotherapy (Kim et al., 2021). Clinical studies have also indicated that selenite can be employed as an anticancer agent with low drug resistance (Brodin et al., 2015). As observed, selenite is more toxic to cancer cells (Figure 3A). Previous studies (Radomska et al., 2021) suggest that cancer cells, characterized by high metabolic activity, tend to absorb more selenium, which may contribute to their higher sensitivity to selenium toxicity, whereas normal cells exhibit lower uptake and are less affected. It has been reported that high ROS levels induce cancer cell death, (Hayes et al., 2020), while low levels of ROS can be used as signal molecules to enhance osteogenic differentiation of MSCs (Bai et al., 2021). Unexpectedly, under the same Se concentration, Cal27 cell viability appeared unchanged (Figure 3F). To determine whether this result reflects inhibited proliferation or induced cell death, we performed apoptosis flow cytometry on Cal27. This analysis revealed a significant increase in both apoptotic and necrotic cells (Figures 6B,C). We suspect that Se suppresses tumours by promoting apoptosis in Cal27 cells. Previous reports show that sodium selenite induces apoptosis in cervical cancer cells via ROS generation and in breast cancer cells through endoplasmic reticulum stress and oxidative stress (Cao et al., 2021; Lv et al., 2024). Our results suggest that Se also promotes apoptosis in HNSCC. Furthermore, high doses of Se compounds inhibit neoplastic growth by producing ROS, (Wallenberg et al., 2014), consistent with our findings (Figure 6A).

The formation of a multi-porous (nano-micron scale) structure on the titanium surface increases surface roughness, alters its hydrophobicity, and provides more binding sites for cells (Jemat et al., 2015; Lorenzetti et al., 2015). Studies have also reported that the pore size of tissue engineering scaffolds can regulate stem cell fate. When the pore size exceeds 250 μm, it promotes the terminal differentiation of BMSCs (Swanson et al., 2021). In our study, even without selenium, the HAMAO coating enhanced ALP expression in rBMSCs (Figure 4C), which may be attributed to its pore size. At the gene expression level, the selenium-containing medium also promoted the osteogenic differentiation of rBMSCs. In the classical signal pathway of MSCs during osteogenic differentiation, BMP2 functions as a pivotal initiating factor that mediates the upregulation of RUNX2 via the BMP/Smad, thereby modulating the expression of downstream osteogenic genes such as COL1 and OPN (Hwang et al., 2023). Ultimately, the progression of osteogenic differentiation can be assessed by evaluating the expression levels of ALP and OCN (Figures 5B–E). It is well known that selenite can exert anti-oxidative stress effects, for instance, by protecting rBMSCs from oxidative stress through activation of the Nrf2 pathway (Rahimi et al., 2023). One potential mechanism involves the Nrf2 (nuclear factor erythroid 2-related factor 2) pathway, a key regulator of oxidative stress. Under high ROS conditions, Nrf2 activation is impaired, leading to reduced antioxidant and increased apoptosis in cancer cells. Conversely, in normal cells such as rBMSCs, controlled ROS levels may act as secondary messengers to promote differentiation via pathways such as Wnt/β-catenin or BMP/Smad signal. Excessive oxidative stress damages osteoblasts, but reducing such stress enhances osteogenesis (Yang et al., 2023). We also found that ROS levels in rBMSCs slightly decreased compared with the control group (Figure 6E), which may protect the cells and further support osteogenesis.

To further establish the clinical potential of this material, the vivo studies are essential to evaluate its long-term biocompatibility, osseointegration, and tumor-suppressive effects in a physiological environment. While our research sheds light on the role of selenium in modulating ROS production and apoptosis, the precise molecular mechanisms driving its osteogenic and antitumor effects remain unclear. Future research should delve deeper into the specific signal pathways involved, which would strengthen the foundation for clinical translation. Addressing these gaps will help advance selenium-modified titanium coatings toward practical applications, providing a dual-functional biomaterial for maxillofacial reconstruction in HNSCC patients.

## 5 Conclusion

In this study, we successfully fabricated a selenium-modified hydroxyapatite coating on titanium using micro-arc oxidation. We verified that this coating possesses favorable surface physical properties, enhances the osteogenic differentiation of MSCs, and promotes the apoptosis of head and neck squamous cell carcinoma cells. Thus, selenium-modified hydroxyapatite titanium coatings offer a promising strategy for repairing jaw defects following tumor surgery. Nonetheless, further investigation is required to elucidate the specific molecular mechanisms and signaling pathways by which selenium exerts its effects, potentially guiding novel therapeutic approaches in the future.

## Data Availability

The original contributions presented in the study are included in the article/supplementary material, further inquiries can be directed to the corresponding authors.
